# Clinical observation of Gofried positive buttress reduction in the treatment of young femoral neck fracture: A systematic review and meta-analysis

**DOI:** 10.1097/MD.0000000000036424

**Published:** 2023-12-01

**Authors:** Huankun Li, Hongjun Chen, Ruihao She, Yanhong Li, Gang Qin, Fukai Gan, Huahui Liang, Baijun Hu

**Affiliations:** a Zhongshan Hospital of Traditional Chinese Medicine Affiliated to Guangzhou University of Chinese Medicine, Zhongshan, Guangdong Province, China; b Shenzhen Traditional Chinese Medicine Hospital, Shenzhen, Guangdong Province, China.

**Keywords:** femoral neck fracture, Gotfried reduction, meta-analysis, nonanatomical reduction, positive buttress

## Abstract

**Background:**

Femoral neck fractures in young adults(<65 years), have always been a difficult problem, characterized by high rates of nonunion and avascular necrosis (AVN). The clinical efficacy of anatomical reduction and non-anatomical reduction methods needs to be supported by clinical data. Therefore, we conduct a meta-analysis on the clinical efficacy of different reduction methods to better guide clinical practice.

**Methods:**

Relevant studies published using internal fixation to treat femoral neck fracture in several databases were searched. The outcomes sought included Harris score and the rate of AVN, nonunion and femoral neck shortening (<5 mm). Included studies were assessed for methodological bias and estimates of effect were calculated. Potential reasons for heterogeneity were explored.

**Results:**

The clinical results showed that compared with the anatomical reduction and positive buttress, there is no significant difference in the rate of AVN (OR = 0.87, 95%CI: 0.55–1.37, *P* = .55), nonunion (OR = 0.54, 95%CI: 0.21–1.41, *P* = .21), femoral neck shortening (<5 mm) (OR = 1.03,95%CI: 0.57–1.86, *P* = .92), the Harris score (MD = −0.28, 95%CI: −1.36–0.80, *P* = .61) and the excellent and good rate of Harris score (OR = 1.73, 95%CI: 0.84–3.56, *P* = .61). However, compared with negative buttress, the rate of AVN (OR = 0.62, 95%CI: 0.38–1.01, *P* = .05), nonunion (OR = 0.34, 95%CI: 0.12–1.00, *P* = .05) and femoral neck shortening (<5 mm) (OR = 0.27, 95%CI: 0.16–0.45, *P* < .00001) were significantly lower, and the Harris score (MD = 6.53, 95%CI: 2.55 ~ 10.51, *P* = .001) was significantly better in positive buttress.

**Conclusions:**

In the case of difficult to achieve anatomical reduction, for young patients (< 65 years) with femoral neck fracture, reduction with positive buttress can be an excellent alternative and negative buttress should be avoided as much as possible.

## 1. Introduction

**Figure 1.**
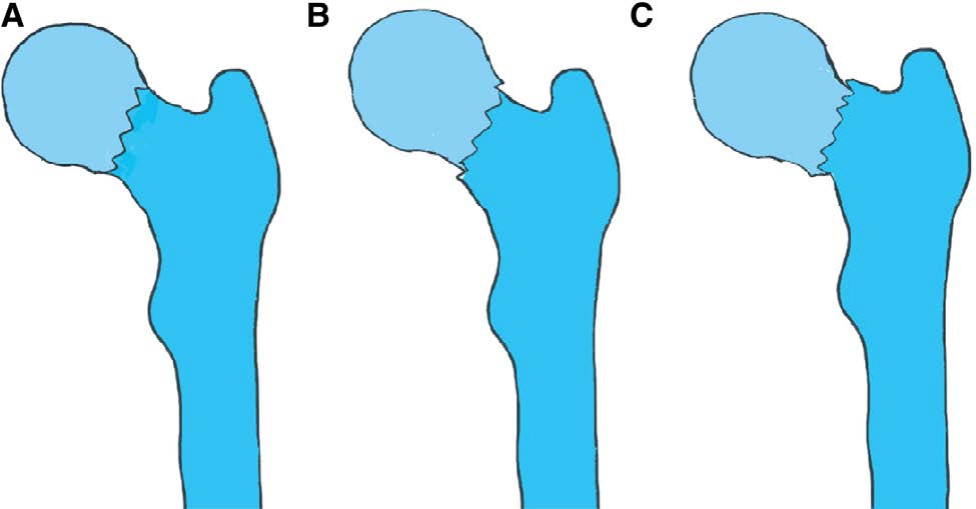
A pattern diagram showing (A) anatomic reduction, (B) positive buttress position reduction in which the distal femoral neck fragment is positioned medially to the lower-medial edge of the proximal fracture fragment, and (C) negative buttress position reduction in which the proximal fracture fragment is displaced medially to the upper medial edge of the distal femoral neck fragment on AP radiograph.

About 4.5 million patients suffer from hip fractures annually, and femoral neck fractures are responsible for a large number of them.^[1]^ Total hip arthroplasty and hemiarthroplasty is becoming a common treatment for elder femoral neck fracture, while in young patients (<65 years), internal fixation is the primary choice considering the longevity of the artificial joint and the risk of second revision.^[2–4]^Due to the destruction of the blood supply, those who treated with internal fixation will suffering from higher rates of complications such as avascular necrosis of the femoral head, nonunion and shortening of femoral neck.^[5–8]^ A meta-analysis published in 2015^[6]^ show that the incidence of isolated femoral neck fractures with reoperation was as high as 18.0%,with avascular necrosis (AVN) was 14.3%, and with nonunion was 9.3%. It remains a challenging issue for orthopedic surgeons. Anatomical reduction (AR) has been considered as the preferred reduction criterion for internal fixation to attain a good prognosis in displaced femoral neck fractures. However, it is usually difficult to achieve anatomical reduction for fractures with obvious displacement, and excessive pursuit of anatomical reduction means further damage to soft tissue vessel,^[9–11]^ which leading to an increase in complication. Thus, in 2013, Gotfried et al^[12]^ firstly proposed the concept of non-anatomical reduction. In anteroposterior (AP) view, the inferior medial edge of the proximal femoral head against the inferior medial edge of the distal (femoral neck) fragment was defined as negative buttress position (NB), while the inferior medial edge of the proximal femoral head against the inferior medial edge of the distal (femoral neck) fragment was defined as positive buttress position (PB) (Fig. 1). They concluded that reduction in positive buttress pattern is accepted when it is difficult to achieve anatomical reduction, but negative buttress pattern should be avoided as far as possible by analyzed the clinical observation of 5 cases.

At present, non-anatomical reduction with Gotfried positive buttress has been recognized by more and more clinicians, and some relevant clinical observation has been reported, but lack of systematic evaluation/meta-analysis of its efficacy. This present study aims to fill this gap for better clinical guidance.

## 2. Methods

### 2.1. Literature search

This study had registered in Prospero (Registration number: CRD42022367354) and was performed in accordance with Preferred Reporting Items for Systematic Reviews and Meta-Analysis guidelines.^[13]^ Base on these guidelines, we searched relevant study in Pubmed, Embase, Cochrane Library, CNKI and WanFang database from 2013 to September 1,2022 (No limit to the language type). The search strategy consisted of medical subject headings(MeSH)”: femoral neck fractures” and text words: “Gotfried reduction,” “Nonanatomical Reduction,” etc. The complete retrieval strategy is shown as “(((((‘Femoral Neck Fractures’[Mesh]) OR (Femoral Neck Fracture)) OR (Femur Neck Fractures)) OR (Femur Neck Fracture)) OR (Subcapital Femoral Fractures)) AND ((((Nonanatomical Reduction) OR (Gotfried reduction)) OR (anatomical reduction)) OR (Non-anatomic reduction))” using PubMed retrieval as an example. The detailed search strategy is as described in the Supplemental Digital Content (see eTable 1–5, http://links.lww.com/MD/K934, http://links.lww.com/MD/K935, http://links.lww.com/MD/K936, http://links.lww.com/MD/K937, http://links.lww.com/MD/K938, Supplemental Contents, that illustrate the search strategy of our study). All study were acquired directly from these databases after reviewed.

### 2.2. Research selection

**Figure 2.**
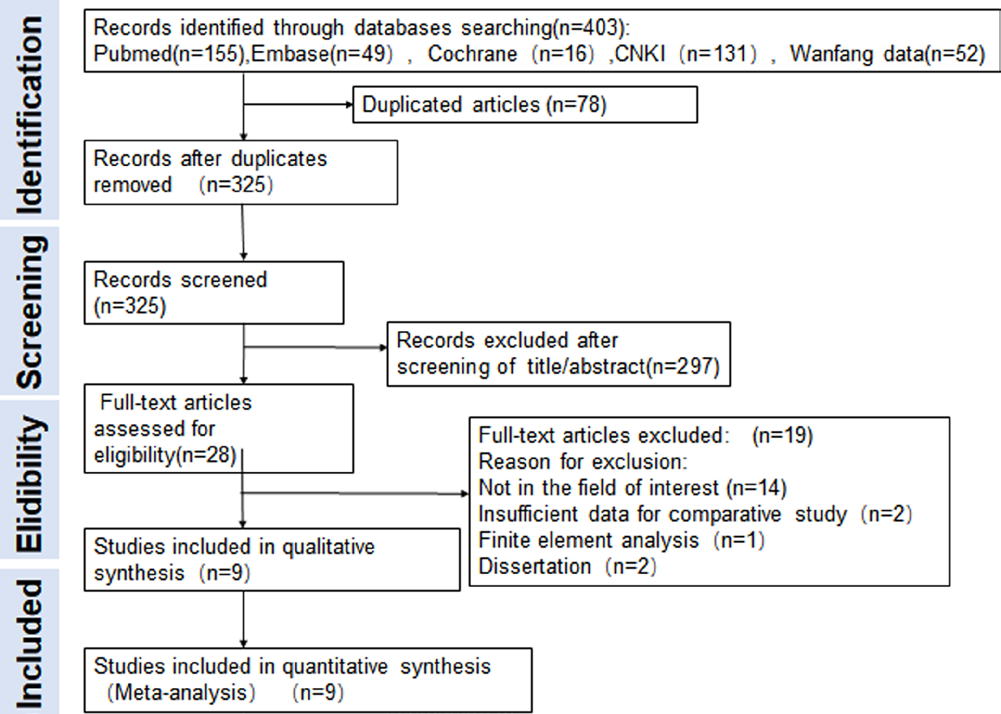
Flow diagram of literature search and study selection. CNKI = China National Knowledge Infrastructure.

Two orthopedic surgeons browse the title and abstract of these studies Independently after entered these retrieval words on the computer. All study records collected in the literature retrieval will be imported to EndNote 9.1 software, and duplicated literatures will be removed. After duplicated literatures were deleted, titles/abstracts were carefully read to evaluate the relevance of other studies. Finally, each orthopedic surgeon assessed for eligibility of remained studies by reviewing full text intensively after removing irrelevance articles. At every stage, any disagreements were settled by consensus through discussion between 2 orthopedic surgeons or seek the help from superior orthopedic surgeon. (Fig. 2).

The inclusion studies were selected by these points as following: Young adult patients with femoral neck fracture(<65 years). Treat with internal fixation. Reduction with anatomical, positive buttress or negative buttress pattern were used to perform the comparison. Randomized controlled trials or non-randomized comparative studies. In addition, the studies will be excluded if they contained one of the following: Case or technical report, reviews, animal experiments, biomechanical and cadaveric studies. Patients were older than 65 years. Records with Insufficient data. Dissertation.

### 2.3. Data extraction and outcome measurement

Two orthopedic surgeons independently reviewed full text of the studies were included. Data was extracted completely by 2 reviewers and any disagreements in data extraction had been settled by discussion and consensus. Baseline data covering the author name, publication year, mean age, sex, type of internal fixation population of different Garden classification and follow-up time were extracted. These data were analyzed to guarantee comparability of each group.

For the meta-analysis, we extracted some typical outcomes such as the rate of AVN, nonunion, shortening of femoral neck (<5 mm), displacement to varus and Harris score.

All of data extracted independently by the same 2 orthopedic surgeons and any disagreements had been resolved by discussion and consensus between the 2 reviewers (Table 1).

**Table 1:** Characteristics of 9 included studies.

Study	Surgery	Positive buttress				Anatomical reduction				Negative buttress				Complications	Harris score
		People (male/female)	Age (years)	Time (months)	Garden classification (I/II/III/IV)	People (male/female)	Age (years)	Time (months)	Garden classification (I/II/III/IV)	People (male/female)	Age (years)	Time (months)	Garden classification (I/II/III/IV)		
Huang 2020	CS	24 (14/10)	48.6 ± 11.3	22.1 ± 11.2	8/6/5/5	21 (12/9)	49.7 ± 11.6	22.4 ± 10.7	7/5/4/5	22 (13/9)	48.3 ± 12.4	22.8 ± 11.6	4/7/5/6	①②③	√
Zhao GL 2019	CS	35	51.4 ± 10.4	27.1 ± 17.6	NC	41	51.4 ± 10.4	27.1 ± 17.6	NC	34	51.4 ± 10.4	27.1 ± 17.6	NC	①②③④	——
Xiong 2021	CS	16 (7/9)	52.4 ± 5.9	20.1 ± 7.2	NC	30 (13/17)	49.2 ± 11.0	22.6 ± 11.7	NC	——	——	——	——	①②③④	——
Zhao G 2021	CS	78 (48/30)	42.3 ± 4.17	49.56 ± 6.96	3/14/33/28	82 (48/32)	42.6 ± 4.33	49.44 ± 8.28	4/13/35/30	62 (36/26)	42.1 ± 4.47	49.32 ± 7.8	2/11/30/19	①②④	√
Zhu 2022	DHS + DS	31 (19/12)	47.71 ± 12.32	47.81 ± 22.22	0/0/22/9	37 (25/12)	51.32 ± 10.24	55.03 ± 24.10	0/0/30/7	——	——	——	——	①②③④	√
Ding 2016	CS	39 (25/14)	47.6 ± 11.7	22.2 ± 10.9	6/5/18/10	40 (29/11)	49.5 ± 11.4	22.1 ± 10.7	5/5/17/13	38 (30/8)	48.7 ± 11.8	22.8 ± 10.7	7/2/17/12	①②	√
Li 2022	PCCP	23 (14/9)	38.24 ± 8.92	12	2/4/8/9	23 (16/7)	36.82 ± 8.73	12	3/3/9/8	23 (17/6)	39.97 ± 9.52	12	3/2/8/10	①②③④	√
Tian 2018	CS	48 (30/18)	29.35 ± 9.85	36	6/8/20/14	48 (32/16)	28.41 ± 9.76	36	4/9/23/12	——	——	——	——	①③④	√
Wang Q 2017	CS	28 (20/8)	47.6 ± 12.1	12	5/3/11/9	33 (21/12)	49.1 ± 10.2	12	4/4/14/11	27 (18/9)	48.1 ± 11.5	12	6/3/10/8	①②③	√

CS = cannulated screws, DHS+DS = dynamic hip screw and derotational screw, NC = not clear, PCC = percutaneous compression plate.

① Rate of AVN; ② Shortening of femoral neck (<5 mm); ③ Displacement to varus; ④Nonunion.

### 2.4. Methodological quality assessment

Considering all of studies were non-randomized trial study, Newcastle-Ottawa scale was recommended to assess the methodological quality and risk of bias of included studies,^[14]^ according to the guideline of Cochrane Handbook.^[15]^ This scale was used to estimated patient selection, comparability, and outcomes that consist of 8 customized assessment sheet criteria. Each criterion is given a score of 1 (star), with the exception of comparability, which is given a score of 2 (star), the higher the score, the higher the quality of the article. The 2 reviewers assessed the quality of included studies independently and disagreements had been resolved by discussion (Table 2).

**Table 2:** Quality assessment of the included studies.

Study	Selections				Comparability	Outcome		
	Representativeness of the intervention cohort	Selection of the non intervention cohort	Ascertainment of intervention	Demonstration that outcome of interest was not present at start of study	Comparability of cohorts on the basis of the design or analysis	Assessment of outcome	Was follow up long enough for outcomes to occur	Adequacy of follow up of cohorts
Huang 2020 (RCS)	☆	☆	☆	——	☆☆	☆	☆	☆
Zhao GL 2019 (RCS)	☆	☆	☆	——	☆☆	☆	☆	☆
Xiong 2021 (RCS)	☆	☆	☆	——	☆☆	☆	☆	☆
Zhao G 2021 (RCS)	☆	☆	☆	——	☆☆	☆	☆	☆
Zhu 2022 (RCS)	☆	☆	☆	——	☆☆	☆	☆	☆
Ding 2016 (RCS)	☆	☆	☆	——	☆☆	☆	☆	☆
Li 2022 (PS)	☆	☆	☆	☆	☆☆	☆	☆	☆
Tian 2018 (RCS)	☆	☆	☆	——	☆☆	☆	☆	☆
Wang Q 2017 (RCS)	☆	☆	☆	——	☆☆	☆	☆	☆
Wang G 2020 (RCS)	☆	☆	☆	——	☆☆	☆	☆	☆

PS = prospective Study, RCS = retrospective cohort studies.

Statement: Our meta-analysis does not address the subject life, health, dignity, privacy, and other related issues. All analyses were based on previous published studies, thus no ethical approval or patient consent was required.

### 2.5. Data synthesis and statistical analyses

For all outcomes, odds ratio (OR) and 95% confidence intervals (CIs) were used to represented dichotomous variables, while mean differences (MD) and 95% CIs represented continuous outcomes. Statistical significance was set at *P* values <.05. The I² statistic and Cochrane Q test were used to assess the heterogeneity of studies, with values of 25%, 50% and 75% were defined as low, medium and high heterogeneity. When I² > 50% or *P* values <.10, the random effect model was used, otherwise the fixed effect model was used. All data were analyzed by RevMan software(version 5.3).^[16]^

## 3. Results

### 3.1. Study identification

The details of study retrieval and selection process are shown in Figure 2. After carefully screening, 9 studies^[17–25]^ were finally included in qualitative synthesis and all for meta-analysis, which consist of 8 retrospective cohort studies (RCS) and 1 prospective study (PS), and represented a total of 883 patient who underwent internal fixation for femoral neck fractures (322 patients underwent internal fixation with reduction in positive buttress pattern, 355 patients underwent internal fixation with anatomical reduction, and 206 patients underwent internal fixation with reduction in negative buttress pattern). Among the 9 studies, 3 studies^[18,21,24]^ simply compared reduction with positive buttress and anatomical reduction and the rest^[17,19,20,22,23,25]^ compared 3 reduction methods. For data recording, dichotomous variables were used for the other outcome indicators included in the comparison, except for the Harris score of 4 articles,^[17,20,22,25]^ which used continuous variables. In the selection of internal fixation, Zhu et al^[21]^ adopted dynamic hip screw and derotational screw (DHS+DS), Li et al^[23]^ adopted percutaneous compression plate (PCCP), and the other studies^[17–20,22,24,25]^ adopted cannulated screws (CS).

### 3.2. Quality assessment

The quality assessment of the included studies were summarized in Table 2. Of all the included studies, 8 were retrospective cohort studies,^[17–22,24,25]^ whose final score was 8 stars with scores were deducted because the clinical outcomes of the included cases were known in advance, while the other scoring indicators were recorded completely. One study^[23]^ was a prospective study with complete data recording and meeting all the criteria of the Newcastle-Ottawa scale, and the final score was 9 stars.

### 3.3. Clinical results

**Figure 3.**
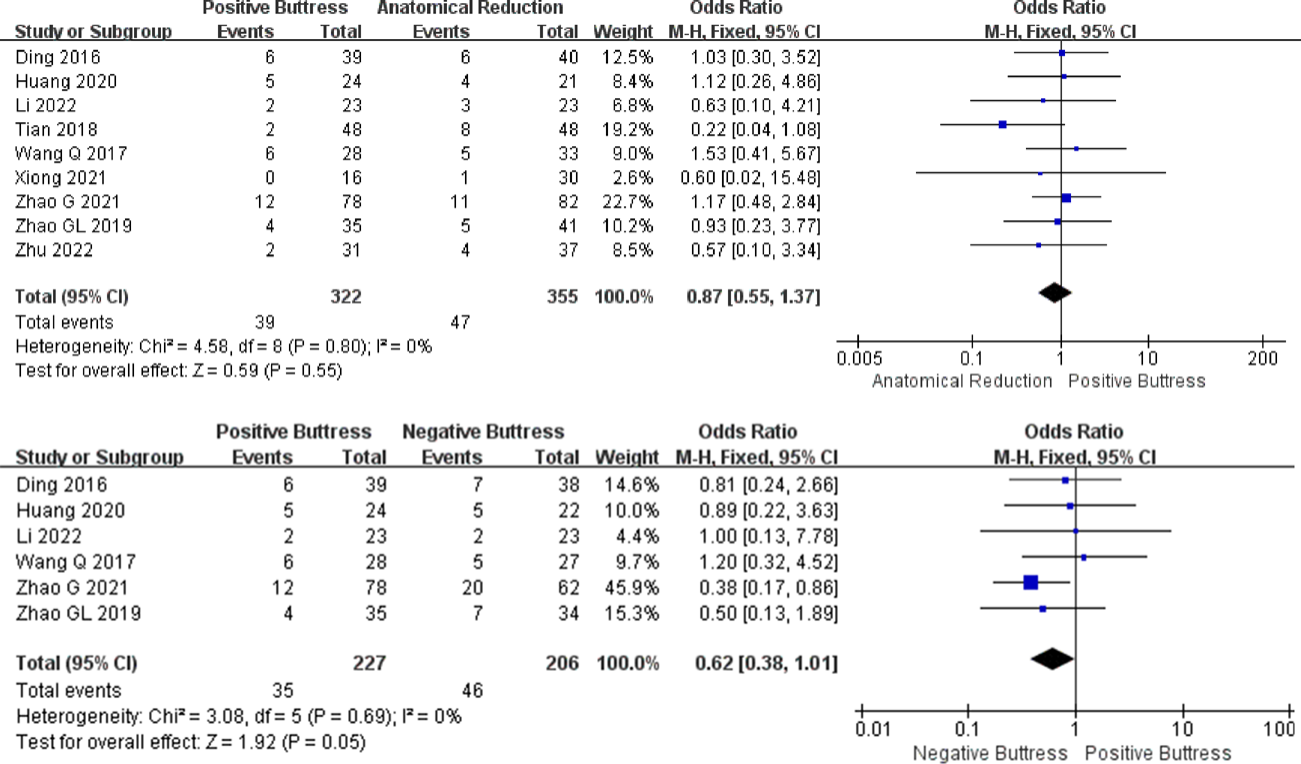
Results of an aggregate analysis comparing the rate of AVN among positive buttress, anatomical reduction and negative buttress. CI = confidence interval.

Nine studies^[17–25]^have reported comparison the between reduction with positive buttress (Group PB, n = 322) and anatomical reduction(Group AR, n = 355) in the rate of AVN. Low heterogeneity among studies (I² = 0%, *P* = .80). The results showed that the rate of AVN in PB group seemed to be lower than that in AR group, but there was no significant difference between the 2 groups (OR = 0.87, 95%CI: 0.55–1.37, *P* = .55). And 6 studies^[17,19,20,22,23,25]^ compared the rate of AVN between reduction with positive buttress (Group PB, n = 227) and reduction with positive buttress (Group PB, n = 206). Low heterogeneity among studies (I² = 0%, *P* = .69). The statistical results showed that the rate of AVN in group PB was lower than that in group NB, and there was a significant difference between the 2 groups (OR = 0.62, 95%CI: 0.38–1.01, *P* = .05). The fixed effect model was used for statistical analysis according to low heterogeneity (Fig. 3).

**Figure 4.**
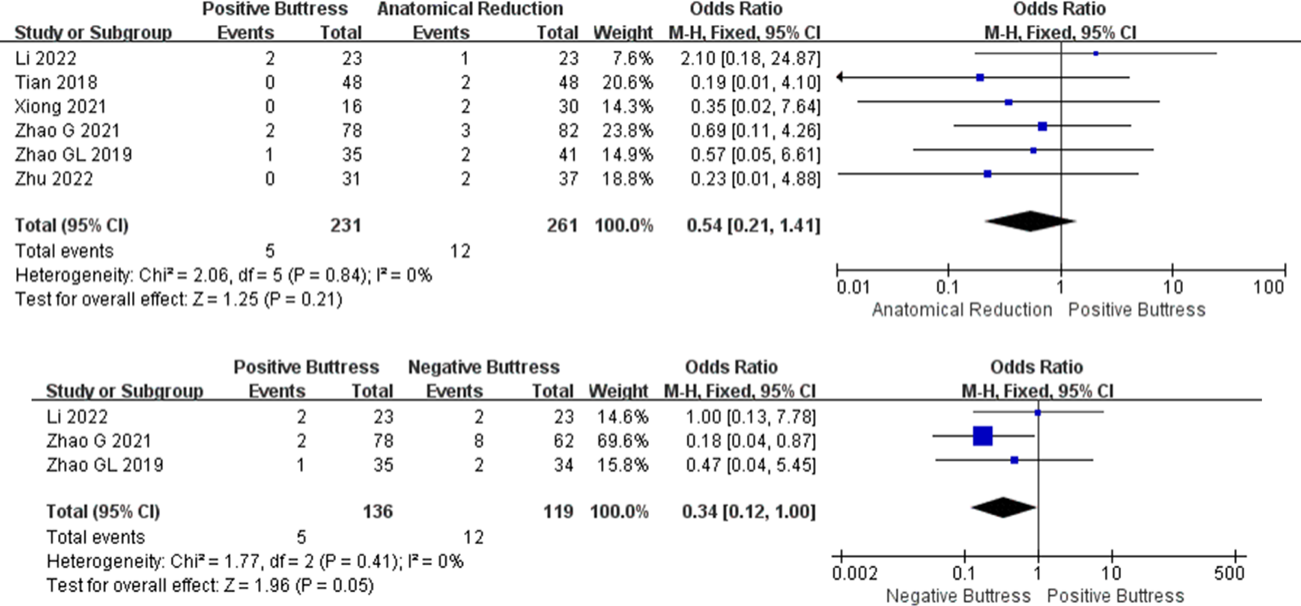
Results of an aggregate analysis comparing the rate of nonunion among positive buttress, anatomical reduction and negative. buttress. CI = confidence interval, SD = standard deviation.

In the incidence of nonunion, 6 studies^[18–21,23,24]^ recorded the comparison between group PB(n = 231) and group AR (n = 261). Low heterogeneity among studies (I² = 0%, *P* = .84), and the fixed effect model was used for statistical analysis. The results show that the incidence of nonunion in group PB was lower than group AR, but there was no significant difference between the 2 groups (OR = 0.54, 95%CI: 0.21–1.41, *P* = .21). However, only 3 studies^[19,20,23]^ compared the incidence of nonunion between the PB group (n = 136) and the NB group (n = 119), with low heterogeneity among the studies (I² = 0%, *P* = .41), and the fixed effect model was used for statistical analysis. The results showed that the incidence of nonunion in the PB group was lower than that in the NB group, and there was a statistical difference between them (OR = 0.34, 95%CI: 0.12–1.00, *P* = .05) (Fig. 4).

Some papers concluded that the distance of postoperative femoral neck shortening with more than 5 mm is closely related to the poor hip function.^[26–28]^Seven of studies^[17–20,22,23,25]^ recorded the degree of femoral neck shortening, according to the indexing method of Xia et al^[28]^ Thus, we select the distance of femoral neck shortening (>5 mm) as an outcome and exclude 1 study^[21]^ from meta analysis because it was not recorded according to this method.

**Figure 5.**
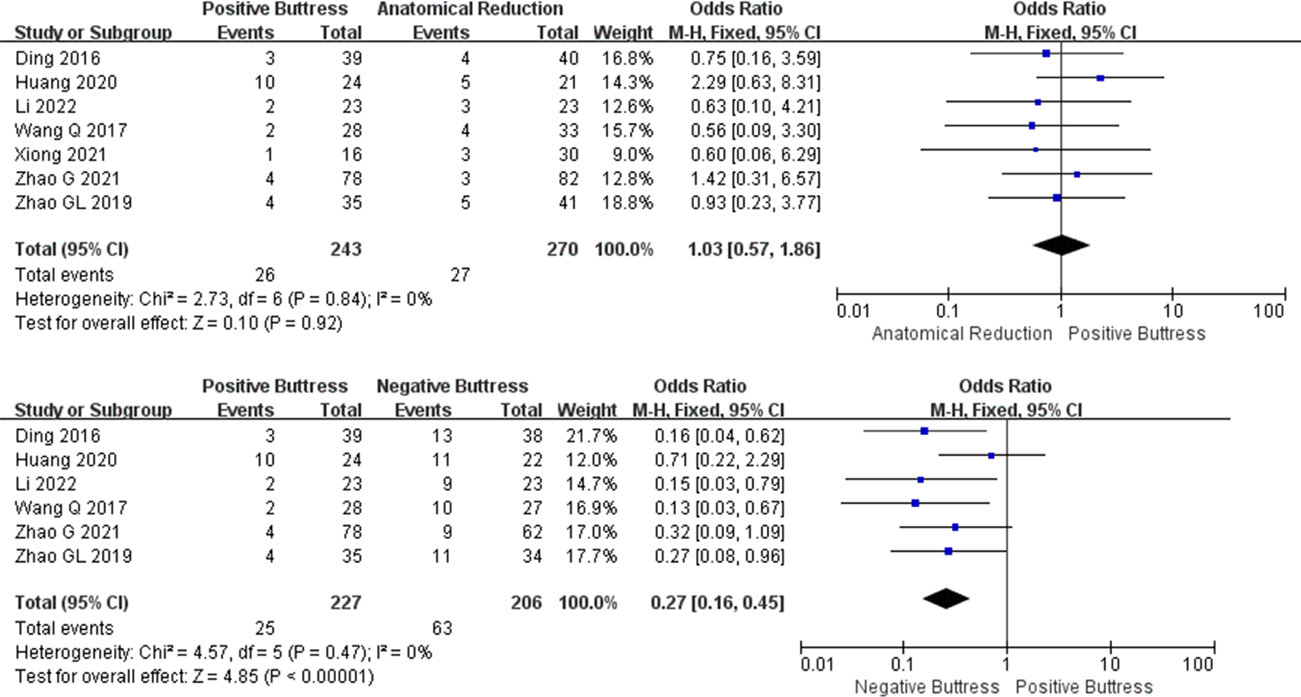
Results of an aggregate analysis comparing the number of Shortening in femoral neck (>5 mm)among positive buttress, anatomical reduction and negative. buttress. CI = confidence interval, SD = standard deviation.

Seven studies^[17–20,22,23,25]^ concluded that there was no significant difference in rate of the femoral neck shortening (>5 mm) between group PB (n = 243) and group AR (n = 270) (OR = 1.03,95%CI: 0.57–1.86, *P* = .92). The fixed effect model was used for statistical analysis for Low heterogeneity (I² = 0%, *P* = .84). And 6 studies^[17,19,20,22,23,25]^ reported the comparison between group PB (n = 227) and group NB (n = 206). The statistical result show that group PB was lower than group NB, and there was a statistical difference between them (OR = 0.27, 95%CI: 0.16–0.45, *P* < .00001). The heterogeneity among the studies was low (I² = 0%, *P* = .47), and the fixed effects model was used for statistical analysis (Fig. 5).

A total of 7 studies^[17,20–25]^ recorded postoperative Harris score, among which 4 literatures^[21,23–25]^ were graded according to the traditional 4 grades of excellent (≥90), good (80~89), and medium (70~79) and poor Harris (<70) score, and the proportion of the number of people who achieved excellent/good rating was selected for statistics. The remaining 3 studies^[17,20,22]^ were recorded by Harris score, and direct statistical comparison was made with continuous variables.

**Figure 6.**
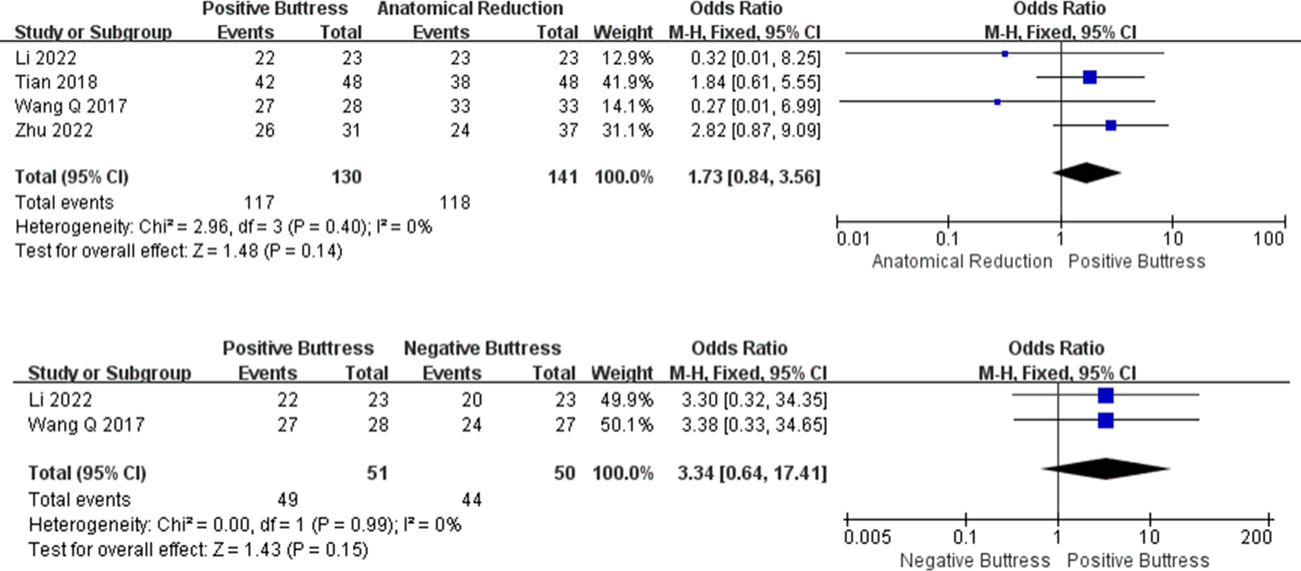
Results of an aggregate analysis comparing the rate (Excellent and Good)of Harris score among positive buttress, anatomical reduction and negative buttress. CI = confidence interval, SD = standard deviation.

In the excellent and good rate comparison of Harris score, group PB (n = 130) was better than group AR (n = 141), but the difference was not statistically significant (OR = 1.73,95%CI: 0.84–3.56, *P* = .14). The heterogeneity among studies was low (I² = 0%, *P* = .40) and the fixed effects model was used for statistical analysis. In addition, only 2 papers^[23,25]^ compared group PB (n = 49) and group NB (n = 44), and the results showed that group PB was better than group NB, but the difference was not statistically significant (OR = 3.34, 95%CI: 0.64–17.41, *P* = .15). The heterogeneity among the studies was low (I² = 0%, *P* = .99), and the fixed effect model was used for statistical analysis (Fig. 6).

**Figure 7.**
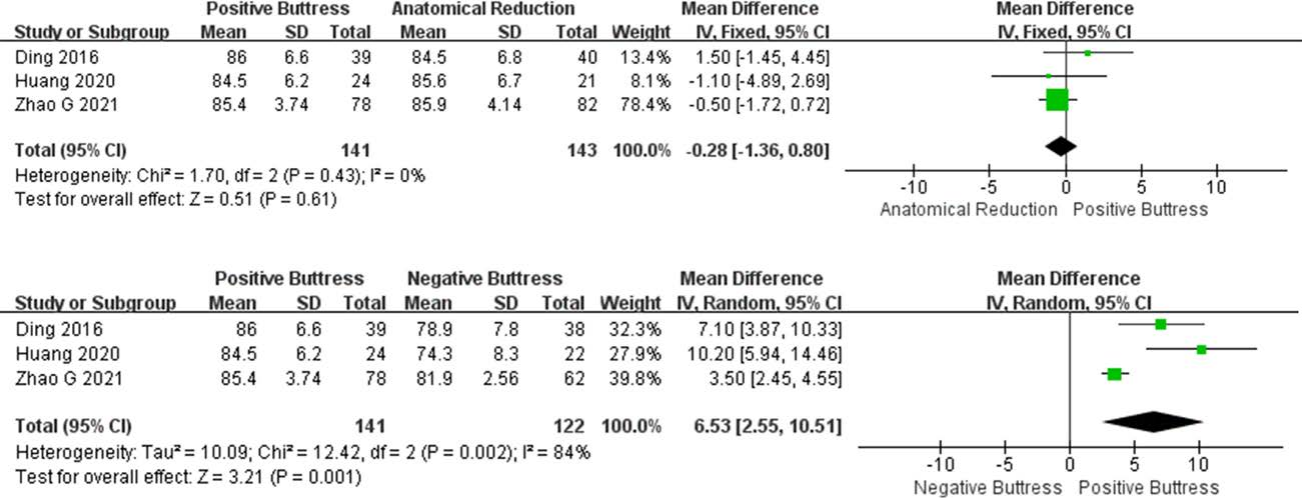
Results of an aggregate analysis comparing the Harris score among positive buttress, anatomical reduction and negative buttress. CI = confidence interval, SD = standard deviation.

In the Harris score comparison, 3 studies^[17,20,22]^ correctly compared group PB (n = 141) and group AR (n = 143) with low heterogeneity (I² = 0%, *P* = .43). Using the fixed effects model, statistical results showed that there was no significant difference between the 2 groups (MD = −0.28, 95%CI: −1.36 to 0.80, P = .61). Three studies^[17,20,22]^ recorded the comparison values between group PB (n = 141) and group NB (n = 122). The heterogeneity among the studies was high (I² = 84%, *P* = .002). The statistical results showed that the PB group was significantly higher than the NB group (MD = 6.53, 95%CI: 2.55 ~ 10.51, *P* = .001), with statistically significant differences. To analyze the cause of high heterogeneity, this outcome was considered as subjective score. Subgroup analyses were not required due to the small number of included studies (Fig. 7).

## 4. Discussion

Necrosis of the femoral head and nonunion are 2 major complications. The univariate analysis of risk factors show that the fracture type, reduction quality, internal fixation mode, Body Mass Index(BMI), ASA Grade are the risk factors for the femoral head necrosis in patients with femoral neck fractures.^[29]^For elderly (>65 years) and patients with femoral neck fractures with low exercise needs, total hip arthroplasty or hemiarthroplasty may be used. Considering the life of prosthetic joints and the risk of revision, necrosis of the femoral head and nonunion should be avoided as much as possible.^[30]^A promising result of reduction is an important indicator of reducing postoperative complications. Although numerous studies have emphasized the importance of anatomical reduction,^[31–33]^ in 2013, Gotfried proposed a criterion of non-anatomical reduction for positive buttress which providing us a new direction.^[12]^

At present, there are 2 main classification methods for femoral neck fracture: Garden classification and Pauwels classification.^[2]^ The Pauwels classification, which is the first biomechanical classification for femoral neck fractures focuses on the shear force,^[34]^ which is closely related to Gotfried positive support theory. Most of the papers included in this study adopted Garden classification, and only 3 of them added Pauwels classification. Now, there is still a lack of clinical efficacy comparison of positive support under different Pauwels classification.

In this study, basic information in the literature such as age, sex, and time of follow-up were included and recorded in detail, as well as important risk factors such as fracture classification methods and internal fixation mode. The statistical results showed that the PB group had no evidence for a statistically significant difference compared with the AR group in AVN and nonunion rate, the loss of postoperative femoral neck length > 5 mm and Harris hip scores, even slightly better than the AR group. Compared with the NB group, the results of each group were significantly better than those of the NB group, with a statistically significant difference. Statistical analysis exhibits low levels of heterogeneity and good comparability among the studies in each group, although the difference in the selection of internal fixation in 2 of the^[21,23]^ studies contributes to heterogeneity. The statistical results are consistent with the views put forward by Gotfried.^[12]^

At present, it is believed that the loss of femoral neck length > 5 mm and the varus angulation of the femoral neck > 10° are closely related to the adverse outcome in femoral neck fracture,^[8,35]^ and fracture type, posterior medial cortex comminution, and reduction quality were the main risk factors for neck shortening.^[36]^ After reading articles, however, which have recorded the incidence rate of coxa vara,^[17–19,21,23–25]^ the team found that the records and criteria for the varus angle were still unclear and controversial. Finally, the meta-analysis of this indicators was excluded. Separate reading found that the comparison results of the 3 reduction methods were essentially in agreement, which showed no statistically significant differences between group PB and group AR. In addition, the varus angle were lower than those of group NB, but it was not possible to determine if these outcomes were statistically significant.

The internal fixation method of femoral neck fracture mainly adopts sliding hip screw constructs.^[37,38]^ Under gravity, the femoral head will slide along the long axis of internal fixation to the base of the femoral neck due to the existence of the femoral neck-shaft angle, thus it would play a role in compressing, fixing and promoting bone healing. But, it is because that the anti-shear force is extremely limited that premature weight bearing is easy to lead to internal fixation cut out from the anterior-upper part of the femoral head, resulting in internal fixation failure and prolonging bed time, which is not conducive to fracture healing.^[39]^ Although the design concept of internal fixation is constantly improved, and femoral neck system (FNS), which has received much attention recently and shows better biomechanical effects,^[40]^ this does not solve the problem of shear force. The positive buttress reduction can provide the support effect of the cortical bone at the basilar part of the femoral neck on the femoral head. It not only can effectively reduce the influence of shear force on fracture broken end, but also can promote bone healing of the lower cortical bone and reduce the contact stress between the femoral head and the internal fixation, thereby lessening the possibility of internal fixation cutout from the femoral head. Wang G and Wang B, et al^[41]^ demonstrated, using animal studies, that Gotfried positive buttress can promote bone repair by promoting angiogenesis and osteogenesis. Compared with these 2 reduction methods, negative buttress reduction does not provide this support effect that the anti-shear force is greatly reduced, which increases the risk of necrosis of the femoral head and nonunion.^[42]^ This is consistent with our statistical results.

Some finite element analyses^[21,43]^ in recent years have verified the better biomechanical effects provided by Gotfried positive buttress reduction. Among them, Wang et al^[43]^ performed the further quantitative analysis the support ability of the positive buttress and suggest that the positive buttress range should be controlled within 3 mm in order to provide effective support. Beyond this limit, the support effect will be greatly reduced. Ghayoumi et al^[44]^ compared open and closed reduction and found that there was no significant difference between the incidence of bone nonunion and AVN while the closed reduction was more dominant in postoperative infection rate, bleeding volume and operation time, even if it is difficult to ensure anatomical reduction. Postoperative femoral head necrosis and bone nonunion in femoral neck fractures are mainly related to the destruction of blood supply,^[45]^ and anatomical reduction does not guarantee a good prognosis. On the contrary, in most cases, especially in some femoral neck fractures that are difficult to reset, repeated adjustments are often required to achieve anatomical reduction regardless of open or not, resulting in further damage to soft tissues and blood vessels, and increasing the possibility of AVN and nonunion.^[44]^ Combined with the results from the aforementioned studies, Gotfried positive buttress reduction has the same efficacy as anatomical reduction in reducing postoperative complications and improving hip function, and theoretically the operation time is relatively shorter. Therefore, in the case of difficult to achieve anatomical reduction, for young patients (< 65 years old) with internal fixation for femoral neck fracture, in order to avoid further iatrogenic injury caused by repeated reduction or open reduction, Gotfried positive support can be achieved and negative support should be avoided as much as possible.

There are also limitations to this study: the patients selected in this study are all Asian and the sample size and scope are relatively small that the further studies including other ethnic groups with larger samples are needed; the information of the surgical doctors is not recorded in detail, which may lead to heterogeneity due to the different surgical experience of different operators; a detailed comparison of the 3 reduction methods in different fracture types. has not been performed and it is hoped that there will be more further research in this regard; the included study was a retrospective cohort study while more high-quality large-sample randomized controlled trials and prospective studies were needed for further validation.

## Author contributions

**Conceptualization:**Huankun Li, Baijun Hu.

**Investigation:** Huankun Li, Hongjun Chen, Ruihao She.

**Methodology:** Huankun Li, Baijun Hu.

**Software:** Huankun Li, Hongjun Chen, Yanhong Li, Gang Qin.

**Supervision:** Baijun Hu, Fu-Kai Gan, Hua-Hui Liang.

**Validation:** Huankun Li, Hongjun Chen, Ruihao She.

**Visualization:** Huankun Li, Hongjun Chen, Yanhong Li, Gang Qin.

**Writing – original draft:** Huankun Li, Hongjun Chen, Ruihao She.

**Writing – review & editing:** Baijun Hu, Fu-Kai Gan, Hua-Hui Liang.

## Supplementary Material











## References

[1] Bhandari M Swiontkowski M. Management of acute hip fracture. N Engl J Med. 2017;377:2053–62. 29166235 10.1056/NEJMcp1611090

[2] Florschutz AV Langford JR Haidukewych GJ. Femoral neck fractures: current management. J Orthop Trauma. 2015;29:121–9. 25635363 10.1097/BOT.0000000000000291

[3] Kang JS Moon KH Shin JS. Clinical results of internal fixation of subcapital femoral neck fractures. Clin Orthop Surg. 2016;8:146–52. 27247738 10.4055/cios.2016.8.2.146 PMC4870316

[4] Biz C Tagliapietra J Zonta F. Predictors of early failure of the cannulated screw system in patients, 65 years and older, with non-displaced femoral neck fractures. Aging Clin Exp Res. 2020;32:505–13. 31677126 10.1007/s40520-019-01394-1

[5] Medda S Snoap T Carroll EA. Treatment of young femoral neck fractures. J Orthop Trauma. 2019;33(Suppl 1):S1–6. 10.1097/BOT.0000000000001369 30540665

[6] Slobogean GP Sprague SA Scott T. Complications following young femoral neck fractures. Injury. 2015;46:484–91. 25480307 10.1016/j.injury.2014.10.010

[7] Xu DF Bi FG Ma CY. A systematic review of undisplaced femoral neck fracture treatments for patients over 65 years of age, with a focus on union rates and avascular necrosis. J Orthop Surg Res. 2017;12:28. 28187745 10.1186/s13018-017-0528-9 PMC5301374

[8] Stockton DJ Lefaivre KA Deakin DE. Incidence, magnitude, and predictors of shortening in young femoral neck fractures. J Orthop Trauma. 2015;29:e293–298. 26226462 10.1097/BOT.0000000000000351

[9] Zhuang L Wang L Xu D. Anteromedial femoral neck plate with cannulated screws for the treatment of irreducible displaced femoral neck fracture in young patients: a preliminary study. Eur J Trauma Emerg Surg. 2019;45:995–1002. 29909465 10.1007/s00068-018-0972-1

[10] Su Y Chen W Zhang Q. An irreducible variant of femoral neck fracture: a minimally traumatic reduction technique. Injury. 2011;42:140–5. 20570257 10.1016/j.injury.2010.05.008

[11] Alizade C Jafarov A Alizada F. Efficiency of an implant: new criterion of objective assessment of implants for osteosynthesis of femoral neck fracture. Int Orthop. 2020;44:569–75. 31848657 10.1007/s00264-019-04439-2

[12] Gotfried Y Kovalenko S Fuchs D. Nonanatomical reduction of displaced subcapital femoral fractures (Gotfried reduction). J Orthop Trauma. 2013;27:e254–9. 23481921 10.1097/BOT.0b013e31828f8ffc

[13] Moher D Shamseer L Clarke M. Preferred reporting items for systematic review and meta-analysis protocols (PRISMA-P) 2015 statement. Syst Rev. 2015;4:1. 25554246 10.1186/2046-4053-4-1 PMC4320440

[14] The Newcastle-Ottawa Scale (NOS) for assessing the quality of nonrandomised studies in meta-analyses. Ottawa Hospital Research Institute; 2010. Available at: https://www.ohri.ca//programs/clinical_epidemiology/oxford.asp [access date 10 September, 2022].

[15] Cochrane Handbook for Systematic Reviews of Interventions, Version 5.1.0. London: The Cochrane Collaboration, 2011. Available at: http://www.cochrane-handbook.org/ [access date 10 September, 2022].

[16] Egger M Davey Smith G Schneider M. Bias in meta-analysis detected by a simple, graphical test. BMJ. 1997;315:629–34. 9310563 10.1136/bmj.315.7109.629 PMC2127453

[17] Huang K Fang X Li G. Assessing the effect of Gotfried reduction with positive buttress pattern in the young femoral neck fracture. J Orthop Surg Res. 2020;15:511. 33160395 10.1186/s13018-020-02039-0 PMC7648971

[18] Xiong WF Chang SM Zhang YQ. Inferior calcar buttress reduction pattern for displaced femoral neck fractures in young adults: a preliminary report and an effective alternative. J Orthop Surg Res. 2019;14:70. 30819226 10.1186/s13018-019-1109-x PMC6396447

[19] Zhao G Liu C Chen K. Nonanatomical reduction of femoral neck fractures in young patients (≤65 years old) with internal fixation using three parallel cannulated screws. Biomed Res Int. 2021;2021:3069129. 33490267 10.1155/2021/3069129 PMC7801101

[20] Zhao G Liu M Li B. Clinical observation and finite element analysis of cannulated screw internal fixation in the treatment of femoral neck fracture based on different reduction quality. J Orthop Surg Res. 2021;16:450. 34256786 10.1186/s13018-021-02580-6 PMC8276405

[21] Zhu J Li Y Zhang Y. Clinical outcome and biomechanical analysis of dynamic hip screw combined with derotation screw in treating displaced femoral neck fractures based on different reduction qualities in young patients (≤65 years of age). Biomed Res Int. 2022;2022:9505667. 35036442 10.1155/2022/9505667 PMC8754672

[22] Shuchen D Rongbin Y Yunlin G. Short-term effectiveness of the Gotfried positive buttress reduction plus fixation with cannulated screws for femoral neck fracture in young and middle-aged people. Chin J Orthop Trauma. 2016;18:655–61.

[23] Lin-tao L Rong-fu H Ying-bing W. Analysis of hip joint function, biomechanics and complications of gotfried positive support reduction combined with percutaneous compression plate for treatment of femoral neck fractures in young and middle-aged people. Heilongjiang Med J. 2022;46:648–51.

[24] Lisheng T. Long-term follow-up study of anatomical reduction and Gotfried support reduction for young femoral neck fracture. Mod Med J China. 2018;20:26–9.

[25] Qi W Gao B Yu Z. One-year follow-up of Gotfried positive buttress reduction combined with internal fixation for treatment of femoral neck fracture. Chin J Pract Med. 2017;44:35–8.

[26] Weil YA Khoury A Zuaiter I. Femoral neck shortening and varus collapse after navigated fixation of intracapsular femoral neck fractures. J Orthop Trauma. 2012;26:19–23. 21904227 10.1097/BOT.0b013e318214f321

[27] Zlowodzki M Ayeni O Petrisor BA. Femoral neck shortening after fracture fixation with multiple cancellous screws: incidence and effect on function. J Trauma. 2008;64:163–9. 18188116 10.1097/01.ta.0000241143.71274.63

[28] Xi X Zhi L. Measurement of femoral neck shortening after cannulated screwing for elderly femoral neck fractures and the effect of shortening on hip function. Chin J Orthop Trauma. 2014;16:651–5.

[29] Pei F Zhao R Li F. Osteonecrosis of femoral head in young patients with femoral neck fracture: a retrospective study of 250 patients followed for average of 75 years. J Orthop Surg Res. 2020;15:238. 32600432 10.1186/s13018-020-01724-4 PMC7322831

[30] Mathews V Cabanela ME. Femoral neck nonunion treatment. Clin Orthop Relat Res. 2004:57–64. 10.1097/00003086-200402000-00010 15021132

[31] Stacey SC Renninger CH Hak D. Tips and tricks for ORIF of displaced femoral neck fractures in the young adult patient. Eur J Orthop Surg Traumatol. 2016;26:355–63. 26965005 10.1007/s00590-016-1745-3

[32] Slobogean GP Stockton DJ Zeng B. Femoral neck fractures in adults treated with internal fixation: a prospective multicenter Chinese cohort. J Am Acad Orthop Surg. 2017;25:297–303. 28248692 10.5435/JAAOS-D-15-00661

[33] Halvorson J. Reduction Techniques for Young Femoral Neck Fractures. J Orthop Trauma. 2019;33(Suppl 1):S12–9. 10.1097/BOT.0000000000001370 30540667

[34] Shen M Wang C Chen H. An update on the Pauwels classification. J Orthop Surg Res. 2016;11:161. 27955672 10.1186/s13018-016-0498-3 PMC5154085

[35] Sun H Shu LY Sherrier MC. Decreased complications but a distinctive fixation loosening mechanism of fully threaded headless cannulated screw fixation for femoral neck fractures in young adults. J Orthop Surg Res. 2021;16:234. 33785020 10.1186/s13018-021-02335-3 PMC8008647

[36] Zhao F Guo L Wang X. Analysis on risk factors for neck shortening after internal fixation for Pauwels II femoral neck fracture in young patients. Eur J Med Res. 2021;26:59. 34167592 10.1186/s40001-021-00531-9 PMC8223273

[37] Duffin M Pilson HT. Technologies for young femoral neck fracture fixation. J Orthop Trauma. 2019;33(Suppl 1):S20–6. 10.1097/BOT.0000000000001367 30540668

[38] Huang S Wang B Zhang X. High-purity weight-bearing magnesium screw: translational application in the healing of femoral neck fracture. Biomaterials. 2020;238:119829. 32058868 10.1016/j.biomaterials.2020.119829

[39] Liu J Zhang B Yin B. Biomechanical evaluation of the modified cannulated screws fixation of unstable femoral neck fracture with comminuted posteromedial cortex. Biomed Res Int. 2019;2019:2584151. 31360707 10.1155/2019/2584151 PMC6642775

[40] Teng Y Zhang Y Guo C. Finite element analysis of femoral neck system in the treatment of Pauwels type III femoral neck fracture. Medicine (Baltimore). 2022;101:e29450. 35839002 10.1097/MD.0000000000029450 PMC11132412

[41] Wang G Wang B Wu X. Gotfried positive reduction promotes the repair of femoral neck fracture potentially via enhancing osteogenesis and angiogenesis. Biomed Pharmacother. 2020;123:109801. 31901717 10.1016/j.biopha.2019.109801

[42] Zhang YQ Chang SM. Mechanism of “Gotfried reduction” in femoral neck fracture. J Orthop Trauma. 2013;27:e291. 10.1097/BOT.0000000000000007 24088775

[43] Wang G Wang B Tang Y. A quantitative biomechanical study of positive buttress techniques for femoral neck fractures: a finite element analysis. Chin Med J (Engl). 2019;132:2588–93. 31658158 10.1097/CM9.0000000000000490 PMC6846255

[44] Ghayoumi P Kandemir U Morshed S. Evidence based update: open versus closed reduction. Injury. 2015;46:467–73. 25554424 10.1016/j.injury.2014.10.011

[45] Ganz R Gill TJ Gautier E. Surgical dislocation of the adult hip a technique with full access to the femoral head and acetabulum without the risk of avascular necrosis. J Bone Joint Surg Br. 2001;83:1119–24. 11764423 10.1302/0301-620x.83b8.11964

