# Supplementary material for: Clinical observation of Gofried positive buttress reduction in the treatment of young femoral neck fracture: A systematic review and meta-analysis
Source: Medicine (Baltimore). 2023 Dec 1;102(48):e36424. doi: 10.1097/MD.0000000000036424 (PMC10695552; doi:10.1097/MD.0000000000036424)
Supplement: Supplementary file 1 [file medi-102-e36424-s001.doc]

eTable 1. The search strategy and results of PubMed

| Serach | Query | Items found |
| --- | --- | --- |
| #1 | ((((("Femoral Neck Fractures"[Mesh]) OR (Femoral Neck Fracture)) OR (Femur Neck Fractures)) OR (Femur Neck Fracture)) OR (Subcapital Femoral Fractures) AND (2013:2022[pdat])) | 7318 |
| #2 | (((((Nonanatomical Reduction) OR (Gotfried reduction)) OR (anatomical reduction)) OR (Non-anatomic reduction)) AND (2013:2022[pdat])) | 9324 |
| #3 | #1 AND #2 | 155 |
