# Supplementary material for: Clinical observation of Gofried positive buttress reduction in the treatment of young femoral neck fracture: A systematic review and meta-analysis
Source: Medicine (Baltimore). 2023 Dec 1;102(48):e36424. doi: 10.1097/MD.0000000000036424 (PMC10695552; doi:10.1097/MD.0000000000036424)
Supplement: Supplementary file 2 [file medi-102-e36424-s002.doc]

eTable 2. The search strategy and results of Embase

| Serach | Query | Items found |
| --- | --- | --- |
| #1 | ‘femoral neck fractures’/exp | 13984 |
| #2 | ‘femoral neck fracture’ | 14576 |
| #3 | ‘femur neck fractures’ | 450 |
| #4 | ‘femur neck fracture’ | 394 |
| #5 | ‘subcaptial femoral fractures’ | 25 |
| #6 | ‘nonanatomical reduction’ | 12 |
| #7 | ‘gotfried reduction’ | 7 |
| #8 | ‘anatomical reduction’ | 1534 |
| #9 | ‘non-anatomical reduction’ | 21 |
| #10 | #1 OR #2 OR #3 OR #4 OR #5 | 14634 |
| #11 | #6 OR #7 OR #8 OR #9 | 1559 |
| #12 | #10 AND #11 AND [2013-2022]/py | 49 |
