# Supplementary material for: Clinical observation of Gofried positive buttress reduction in the treatment of young femoral neck fracture: A systematic review and meta-analysis
Source: Medicine (Baltimore). 2023 Dec 1;102(48):e36424. doi: 10.1097/MD.0000000000036424 (PMC10695552; doi:10.1097/MD.0000000000036424)
Supplement: Supplementary file 3 [file medi-102-e36424-s003.doc]

eTable 3. The search strategy and results of the cochrane library

| Serach | Query | Items found |
| --- | --- | --- |
| #1 | MeSH descriptor :[Femoral Neck Fractures] explode all trees | 467 |
| #2 | (Femoral Neck Fracture):ti,ab,kw | 2534 |
| #3 | (Femur Neck Fractures):ti,ab,kw | 1237 |
| #4 | (Femur Neck Fracture):ti,ab,kw | 1237 |
| #5 | (Subcaptial Femoral Fractures):ti,ab,kw | 45 |
| #6 | (Nonanatomical Reduction):ti,ab,kw | 5 |
| #7 | (Gotfried Reduction):ti,ab,kw | 1 |
| #8 | (Anatomical Reduction):ti,ab,kw | 1501 |
| #9 | (Non-anatomical Reduction):ti,ab,kw | 7 |
| #10 | #1 OR #2 OR #3 OR #4 OR #5 | 2838 |
| #11 | #6 OR #7 OR #8 OR #9 | 1501 |
| #12 | #10 AND #11 | 16 |
