# Supplementary material for: Clinical observation of Gofried positive buttress reduction in the treatment of young femoral neck fracture: A systematic review and meta-analysis
Source: Medicine (Baltimore). 2023 Dec 1;102(48):e36424. doi: 10.1097/MD.0000000000036424 (PMC10695552; doi:10.1097/MD.0000000000036424)
Supplement: Supplementary file 4 [file medi-102-e36424-s004.doc]

eTable 4. The search strategy and results of CNKI (2013.01.01-2022.09.01)

| Serach | Query | Items found |
| --- | --- | --- |
| #1 | TKA=(股骨颈骨折) | 10035 |
| #2 | TKA=(阳性支撑+正性支撑+Gotfried复位+阳性复位+非解剖复位+解剖复位) | 3672 |
| #3 | #1 AND #2 | 131 |
