# Supplementary material for: Clinical observation of Gofried positive buttress reduction in the treatment of young femoral neck fracture: A systematic review and meta-analysis
Source: Medicine (Baltimore). 2023 Dec 1;102(48):e36424. doi: 10.1097/MD.0000000000036424 (PMC10695552; doi:10.1097/MD.0000000000036424)
Supplement: Supplementary file 5 [file medi-102-e36424-s005.doc]

eTable 5. The search strategy and results of Wanfang database. (2013-2022)

| Serach | Query | Items found |
| --- | --- | --- |
| #1 | 题名或关键词:("股骨颈骨折") | 13785 |
| #2 | 题名或关键词:("阳性支撑" or "Gotfried复位" or "正性支撑" or "阳性复位" or "非解剖复位" or "解剖复位") | 1795 |
| #3 | #1 AND #2 | 52 |
